# Cohort Profile for the Heat in Pregnancy- India (HiP-India) Study

**DOI:** 10.12688/wellcomeopenres.24393.2

**Published:** 2025-11-17

**Authors:** Ramachandran Thiruvengadam, Sreevatsan Raghavan, Rekha Shanmugam, Tanya Isaac, Gabriela De Jesus Cipriano Flores, Arya Thonikund Sathishkumar, Sudhakar Reddy Bulla, Prakriti Dayal, Ayushi, Mudita Gosain, Neera Parmar, Lovejeet Kaur, Divya Arya, Savita Singh, Sumit Misra, Dharmendra Sharma, Taruna Rattan, Mukesh Juyal, Sunil Kumar Nanda, Mary Daniel, Padma Alaganandam, Rachna Jain, Prashanth Nigam, Shantanu Pathak, Gabriel Jones, Fadil M. Hannan, Koundinya Desiraju, Shailaja Sopory, Pallavi Kshetrapal, Stalin Prabakaran, Sangeeta Raman Jogi, Praveen Devarsetty, Alka Singh, Manu Vatish, Basky Thilagnathan, Sagnik Dey, Vidhya Venugopal, Ashok Khurana, Aris T Papageorghiou, Yogesh Jain, Mark Woodward, Jane E Hirst, Shinjini Bhatnagar, Nitya Wadhwa, Heat in Pregnancy-India consortium

**Affiliations:** 1 Translational Health Science and Technology Institute, Faridabad, Haryana, 121001, India; 2 Pondicherry Institute of Medical Sciences, Puducherry, Puducherry, 605014, India; 3 Sri Ramachandra Institute of Higher Education and Research (Deemed to be University), Chennai, Tamil Nadu, 600016, India; 4 The George Institute for Global Health UK, Oxford, England, W12 7RZ, UK; 5 The George Institute for Global Health India, New Delhi, Delhi, 110025, India; 6 Chhattisgarh Institute of Medical Sciences, Bilaspur, Chhattisgarh, 495001, India; 7 CareMother, Mumbai, Maharashtra, 411045, India; 8 Nuffield Department of Women's & Reproductive Health, University of Oxford, Oxford, England, OX1 2JD, UK; 9 Chief Medical Officer, Municipal Corporation of Gurugram, Chandigarh, Chandigarh, 160001, India; 10 St George's University Hospitals NHS Foundation Trust, London, England, SW17 0QT, UK; 11 Indian Institute of Technology Delhi, New Delhi, Delhi, 110016, India; 12 Department of Health, Management and Policy, Korea University, Seoul, South Korea; 13 The Ultrasound Lab, New Delhi, Delhi, 110024, India; 14 The George Institute for Global Health, Newtown, New South Wales, Australia

**Keywords:** heat exposure, pregnancy outcomes, maternal health, plancental function, air pollution, fetal development, heat strain, india

## Abstract

**Background:**

Extreme heat exposure — defined as sustained ambient temperatures exceeding local thresholds — has been associated with several adverse pregnancy outcomes, including preterm birth, stillbirth, gestational diabetes and small for gestational age. However, the mechanisms linking environmental heat to these outcomes, and the biological markers that signify individual vulnerability, are not well understood. We present the protocol for a prospective cohort study within the Heat in Pregnancy-India consortium (HiP-India). This study aims to characterise the physiological and pathophysiological responses of the mother, placenta, and fetus to varying levels of temperature, humidity, and air pollution exposure, and to identify the critical windows and mechanisms of heat-related risk during pregnancy.

**Methods:**

600 women with singleton pregnancies, with confirmed gestational age by ultrasound between 11–14 weeks, will be recruited prospectively from three distinct climate zones in India: Gurugram, Delhi NCR ‘semi-arid’; Bilaspur, Chhattisgarh ‘humid sub-tropical and tropical wet and dry’; and Puducherry ‘tropical wet and dry’. Each participant will have their level of exposure to heat, humidity and air pollution measured for 24 hours each trimester in their home and/or workplace using individual and area monitoring devices. Perceived heat stress will be captured using a modified HOTHAPS questionnaire, while physiological heat strain will be measured through urinary specific gravity, core body temperature, heart rate and blood pressure. Within 48 hours of environmental monitoring, maternal haemodynamic parameters will be assessed non-invasively. Fetal ultrasound will be performed to evaluate growth and fetal-placental blood flow, and maternal blood samples collected to evaluate circulating biomarkers of placental function and stress. Cardiotocography will be conducted in the third trimester only. Delivery outcomes for both mothers and neonates will be extracted from hospital records and interviews. In a subset of 100 women, markers of lactation physiology will be recorded during the first 2 weeks after delivery.

**Ethics and dissemination of results:**

All necessary ethical approvals from relevant committees at participating institutions have been obtained. Written informed consent will be obtained from all participants. The findings from this study are expected to inform climate adaptation strategies and emergency response policies to protect pregnant populations from the impacts of extreme heat, both within India and in other similarly affected regions globally. Results are aimed for journal publication, communicate findings to participants in plain language, disseminating information at conferences and events of similar nature.

## Background

Pregnant women residing in tropical climates, particularly from low- and middle-income countries (LMICs) are some of the most vulnerable to the effects of climate change ^1^. Exposure to extreme heat, defined as ambient temperatures exceeding locally adapted thresholds, has been linked to a range of adverse pregnancy outcomes, including preterm birth, stillbirth, gestational diabetes, small for gestational age, and preeclampsia ^2, 3^. These adverse outcomes contribute to infant and under-5 mortality and morbidity, exacerbating existing health inequities in LMIC settings.

India has reported a marked rise in the intensity, frequency, and duration of heatwaves over the last half century ^4^. The years 2022 and 2024 were documented to be the hottest since 1901, as per the records of the Indian Meteorological Department (IMD). Recent studies have revealed a spatiotemporal shift in heat wave events over India, resulting in the identification of three distinct heat wave hotspots in the country: Northwestern, Central, and South-Central India ^4, 5^. Rekha *et al.* have documented the heightened risk of mothers working in high-temperature settings in India with adverse pregnancy outcomes ^6^. However, the biological mechanisms that mediate these effects — including maternal thermoregulation, placental perfusion, and fetal development — remain poorly characterised.

The Heat in Pregnancy-India (HiP-India) consortium aims to understand how extreme heat exposure affects maternal, placental, fetal, and lactation physiology, and how these changes result in adverse pregnancy outcomes, to help identify who is most vulnerable during extreme heat events, and future interventions to protect pregnant women and babies. The consortium is adopting an ambispective study design, utilising both retrospective data (from the GARBH-Ini cohort ^7^, and a large nationally representative, anonymised database of fetal heart rate monitoring), and prospective data from a novel cohort of women recruited from three different climate zones in India. Parallelly, the study will assess women’s experiences and protective mechanisms against extreme heat in the three sites, as part of the qualitative and community engagement part of the research. The retrospective and qualitative branch of the study are not specified in this paper. This manuscript presents the profile and methodology of the prospective cohort arm.

## Objectives

### Study hypothesis

**Figure 1.**
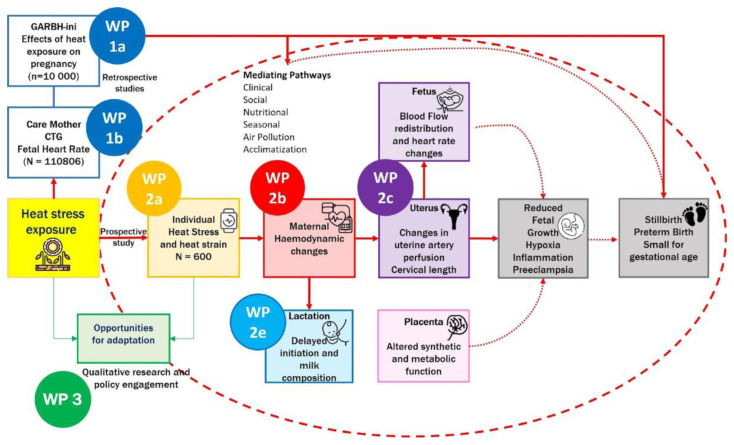
Overall HiP-India Study overview and prospective cohort study highlighted under the red dashed circle. *WP= Work Package. The prospective cohort described in this paper encompasses WP 2a–e*.

It is hypothesised that environmental heat exposure during pregnancy adversely affects maternal, placental, fetal, and lactational physiology through disruptions in haemodynamics, placental function, and thermoregulatory mechanisms. These physiological alterations may lead to measurable changes in biomarkers, fetal growth trajectories, and birth outcomes. Refer to Figure 1.

### Primary objective

To determine the effect of exposure to environmental heat, humidity, and air pollution on maternal, fetal, and placental biological and physiological parameters during pregnancy and identify putative pathways leading to adverse pregnancy outcomes in Indian women.

### Secondary objectives

1. To identify biomarkers and clinical factors associated with heat exposure and adverse pregnancy outcomes.

2. To explore the mechanistic pathways linking environmental heat exposure to adverse pregnancy outcomes.

3. To identify potential confounders and effect modifiers of the associations

## Study design

This will be a multicentre prospective cohort study enrolling pregnant women at 11–14 weeks of gestation and following them up until two weeks after birth. The study aims to capture detailed environmental, clinical, physiological, and biochemical data across key gestational windows, enabling a comprehensive assessment of the maternal and fetal response to heat exposure over time.

### Study setting

The three study locations were selected to represent distinct climate zones across India, each with unique environmental exposures relevant to maternal and child health. Gurugram is located within the Delhi National Capital Region (NCR) and experiences a **semi-arid climate**, characterised by extreme temperature variations, with scorching summers, cold winters, and limited rainfall, resulting in prolonged dry spells and frequent heatwaves. Chhattisgarh represents a **humid subtropical to tropical wet and dry climate**, marked by high humidity, hot summers, and a well-defined monsoon season, creating conditions of alternating intense heat and moisture. Puducherry, situated along the southeastern coast, exemplifies a **tropical wet and dry climate**, with persistently high temperatures, elevated humidity, and pronounced monsoon periods, particularly during the northeast monsoon. These diverse climatic conditions provide a natural contrast in thermal environments, enabling the study to capture a range of heat exposures and their differential impacts on pregnancy-related outcomes across varying ecological contexts.

**Site 1: Gurugram**

Gurugram Civil Hospital (GCH) is a secondary-care hospital that serves a rural and semi-urban population of 1.5 million people in the Gurugram district in Haryana, Northern India. GCH is a referral hub for 22 primary health centres and every month, an average of 900 women attend the antenatal clinics, and 500 infants are delivered in the hospital. Safdarjung Hospital (SJH) is the referral institution for GCH for high-risk pregnancies. The enrolled participants are provided with standard care by the physicians at both GCH and SJH.

**Site 2: Puducherry**

Pondicherry Institute of Medical Sciences (PIMS) is a multi-speciality hospital and teaching Institute in the Union Territory of Puducherry, South India. The hospital is funded by the Madras Medical Mission, a charitable organisation that offers healthcare services at subsidised rates. PIMS laboratory is National Accreditation Board for Testing and Calibration Laboratories (NABL) accredited in medical testing for Clinical Biochemistry, Clinical Pathology, Haematology, Histopathology, Microbiology & others. PIMS has established the Demographic Developmental Environmental Surveillance Site (DDESS) funded by the Department of Biotechnology, Government of India which consists of a population of over 56,000 across 24 villages in the districts of Pondicherry and Tamil Nadu. Approximately 90 women attend the antenatal clinic daily with 100 deliveries conducted monthly. Study participants will be recruited from these villages through community-based screening.

**Site 3: Chhattisgarh**

Chhattisgarh Institute of Medical Sciences is a multispecialty hospital and the second largest public medical college in Bilaspur in Chhattisgarh. The department of Obstetrics and Gynaecology runs a busy clinical service with 70 to 80 women attending each antenatal outpatient clinic, daily admissions of 25 to 30 pregnant women and approximately 600 deliveries each month. The department also draws a large number of emergency referrals from rural areas of the district as well as neighbouring districts. The department of radiodiagnosis performs 15 ultrasound scans per day, with other cases performed in the private sector. Diagnostic services in imaging, biochemistry, pathology, and microbiology are available on-site, with approximately 15 antenatal ultrasound scans performed daily. Study participants will be recruited from the antenatal outpatient clinics at CIMS.

### Inclusion criteria

Pregnant women fulfilling the following inclusion criteria will be considered eligible if they have:

- Confirmed singleton pregnancy
- Gestational age between 11 and 13+6 weeks at the time of recruitment, determined by ultrasound-based crown-rump length measurement
- Recruitment during predefined seasonal windows corresponding to site-specific climatic extremes:
  - Gurugram: warm season (April–July) and cool season (November–February)
  - Puducherry: warm season (March–June) and cool season (November–February)
  - Chhattisgarh: warm season (March–June) and cool season (November–February);
- Elevated risk of heat exposure based on occupational or household factors, including employment in agriculture, construction, factories, or street vending; residence in poorly ventilated homes, homes with metal roofing, or use of traditional biomass stoves (chulhas)
- Willingness and ability to provide informed consent and participate in all study assessments, including home- and workplace-based environmental monitoring throughout pregnancy

### Exclusion criteria

Pregnant women will be excluded if they:

- Are aged below 18 years
- Regular use of fixed air conditioning at home or workplace (use of fans or coolers is permitted)
- Have an underlying severe medical condition (e.g. end-stage renal failure, cardiac disease, cancer, severe mental health problems, severe anaemia, severe endocrine and metabolic disorders)
- Intention to relocate from the study area prior to delivery
- Evidence of active vaginal bleeding at the time of recruitment
- Suspected chromosomal or structural fetal anomalies incompatible with life, identified on first trimester ultrasound

### Recruitment

Potential participants will be identified by clinic staff deployed at each hospital antenatal care centre and community health workers from surrounding villages. Recruitment will be supported by routine screening during antenatal visits, community outreach, and engagement with local health workers to ensure adequate enrollment and timely identification of eligible women to meet the target sample size. The project staff will approach potential participants and provide verbal information about the project, along with the participant information sheet. To supplement the information about the project, the team will provide pamphlets at each site, briefly describing its aims and overall outline, including details about the home visits. For those with low literacy or a preference for oral or visual information, a short video will be shown to convey the same information in the local language, which can be viewed on the project member’s tablet computer. The participant information sheet will describe the project, outline the activities involved if they participate, and detail any potential risks and benefits. Participation in the study will not influence clinical care delivery in each site; however, if clinical conditions are suspected during any of the study assessments, women will be referred to the local obstetric team for review. Participants will have an opportunity to ask questions to the study team members.

An orientation to the study will also be conducted for the landlords for the participants who stay in rented accommodations to explain the nature of the environmental assessments. 

For women who are willing to participate, a dating ultrasound will be arranged within the next 7 days to confirm her pregnancy is (i) viable and the inclusion criteria as mentioned above. If the dating ultrasound report indicates a gestation period of less than 10 weeks, the participant will be invited back for another scan within the enrolment window period of 11- 13+6 weeks of gestation. The participant will be asked to provide written informed consent to participate in the study after confirmation of the inclusion criteria. It will be made clear that she can withdraw her consent at any time during the study, with the option also to remove any data collected up to that point if she wishes. As all participants at the GCH site will also be enrolled in the GARBH-Ini Cohort ^7^, participation in this study will require a separate consent.

The informed consent will include participation for home/workplace climate and heat strain assessments, ultrasound scans conducted at each trimester, maternal haemodynamic assessments, blood tests taken in each trimester, CTG in the third trimester, and for outcomes at birth to be extracted from her medical records and those of her baby either at site (if delivery done at site) or from after delivery through a planned home visit. A subset of women at the GCH site will also be consented for lactation samples and an additional blood sample to measure serum prolactin levels.

### Biospecimen and data collection methods

Each site will have a separate dedicated team of research physicians, study nurses, clinical & laboratory technicians and field workers. These site teams along with site and project managers will form a cumulative clinical research unit (CRU), who would be responsible for implementing all study-related procedures at the three sites.

Sample and data collection at the study sites, during home visits, or by telephone interviews will be conducted by the study nurses using electronic forms under the supervision of medically qualified and trained research physicians. The field workers are responsible for ensuring that participants adhere to the study follow-up schedule. A separate trained research team ensures compliance to follow-up schedules. Local research laboratory teams will collect all biospecimens, pre-processing will be carried out at each of the three study sites respectively and transported to THSTI for long term archival at the ISO20387 accredited biorepository facility following standard operating protocols to ensure sample integrity and quality.

After confirmation of eligibility and recruitment, women will undergo assessments at three time points during pregnancy corresponding with the three trimesters summarised in Table 1 and Table 2 respectively.

**Table 1.** List of procedures and assessments at three time points during pregnancy corresponding with the three trimesters for the study participants.

	Start				Close out	
		N=600	N=600	N=600	N=600	N=100
Timepoint	t _-1_	t _0_ 11–14 weeks	t _1_ 18–22 weeks	t _2_ 30–34 weeks	t _4_ birth	t _5_ 0–16 days PN
**RECRUITMENT**						
Eligibility screen	X					
Informed consent	X					
Gestational age confirmation by ultrasound	X					
Confirmation of viable singleton pregnancy	X					
**ASSESSMENTS**						
Clinical history		X	X	X	X	
Food frequency questionnaire		X	X	X		
Heat exposure and indoor pollution measurements		X	X	X		X
Modified HOTHAPS questionnaire		X	X	X		
Urine specific gravity		X	X	X		
Maternal Haemodynamic assessment		X	X	X		
Fetal Ultrasound		X	X	X		
CTG				X		
Maternal blood sample		X	X	X		
Birth and newborn outcomes					X	
Lactation study (100 women only in GCH)					X	X

**Table 2.** List of planned clinical samples with their processing methods and planned future course of analysis.

The sample collection, processing methods are summarized in Table 4: Biospecimen	Time of collection	Method of collection	Immediate processing at study site	Transportation time to storage at biorepository	Long term storage	Studies*
Maternal Blood -5 ml (EDTA tube)	11–14 weeks 18–22 weeks 30–34 weeks Time of Delivery	Venepuncture	1. Stored as buffy coat 2. Processed to plasma	In liquid nitrogen (-196 ° C) / within 8 hours for GCH site and in Dry Ice for other two sites	Deep freezers (-75 °C)	Biobanking, biochemistry,
Maternal Blood (plain tube) – 5 ml	11–14 weeks 18–22 weeks 30–34 weeks Time of Delivery 15 days post delivery (pre and post feed)	Venepuncture	Processed to sera	In liquid nitrogen (-196 ° C) / within 8 hours for GCH site and in Dry Ice for other. two study sites	Deep freezers (-75 °C)	Biobanking, biochemistry,
Breast milk	First and 15 days post delivery	Manual expression	Stored in 0.5 ml aliquots up to a total volume of 5 ml	In liquid nitrogen (-196 ° C) / within 8 hours for GCH site and in Dry Ice for other. two study sites	Deep freezers (-75 °C)	Biobanking, biochemistry

### Measurement of potential confounders

To adjust for potential confounding factors, comprehensive baseline information will be collected from all participants at enrolment and updated at each subsequent visit. This includes socio-demographic details (e.g., maternal age, education, occupation, household income, type of housing material, and access to cooling resources), medical and obstetric history (e.g., parity, prior adverse pregnancy outcomes, chronic health conditions), and social factors (e.g., marital status, family support, and migration status). Dietary intake will be assessed using site-specific Food Frequency Questionnaires (FFQs), adapted and validated for the local population where possible along with physical activity using the International Physical Activity Questionnaire (IPAC) ^8^. These FFQs will capture habitual intake of major food groups and specific nutrients relevant to pregnancy and heat vulnerability. Housing structure and ventilation characteristics, source of water and sanitation, and presence of cooling appliances (e.g., fans, air conditioners) will also be recorded through structured interviews and home visit observations. These variables will be used in subsequent analyses to adjust for confounding and assess effect modification in the relationship between environmental exposures and maternal or neonatal outcomes.

Within the overall design of the study, expert work groups have been convened to design and oversee the capture of key exposure and outcomes, which are given below. This protocol will outline the aims of each component and methods for sample/data capture.

### Individual environmental exposures

Heat events in this study are defined using three approaches: (1) the India Meteorological Department (IMD) definition of a heatwave; (2) threshold exceedance of wet bulb globe temperature (WBGT) values based on international occupational safety guidelines ^9^; and (3) statistical exceedances, including continuous measures and the 95 ^th^ percentile of local temperature distributions. This multi-pronged definition allows us to capture both acute and chronic heat stress relevant to maternal and foetal outcomes. The full list of terms is given as a glossary at the end of the manuscript.

To determine ambient heat exposure, satellite-based temperature via global positioning system (GPS) coordinates and humidity data, with microenvironmental measurements, will be integrated. For home-based assessments, data will be captured over 24 hours once per trimester using ground-based instruments (EL-USB-2-LCD+ data logger, and iButton Hygrochron) and 4–5 heat exposure measurements using Questemp°34 3M WBGT Monitor. A sub-study with repeated assessments (48–72 hours) will be conducted in ~10% of participants at each site. This will allow estimation of within-and between-day variance, supporting regression calibration or other error-modelling approaches. Additionally, if the woman is working, workplace exposure will be captured during an 8-hour shift (pre-shift, multiple during the shift, and post-shift) each trimester, American Conference of Governmental Industrial Hygienists (ACGIH) screening limits (i.e., preliminary heat exposure thresholds used to identify potential health-related health risks in the workplace) and a professional Industrial Hygienist’s judgement will determine the pregnant woman’s workload category (Heavy or moderate job type). The Threshold Limit Value (TLV) will be calculated by obtaining spot readings during the work shift/working time and by workers/participants describing workload, using a "clo" factor of 0.6 for summer work uniforms. This "clo" component contributes to a WBGT correction factor. Since no such standards exist for the general population, the same ACGIH standards will be applied to the general population as the study participants include both working mothers and non-working mothers who perform moderate to heavy-intensity household activities such as cooking, cleaning, and childcare, making the activity relevant to occupational standards appropriate for our context.

These measurements will be spatially and temporally linked to satellite-derived meteorological data via GPS and timestamps, allowing for triangulation of heat exposure levels, adjustment for spatial variability, and improved resolution. The satellite-based temperature and humidity data retrieved for the entire pregnancy will be incorporated to complement individual-level measurements, supporting a more comprehensive assessment of cumulative heat exposure. By combining home and workplace measurements, this approach holistically captures heat exposure and its health impacts, ensuring a complete assessment of each woman’s complete environmental burden.

Personal heat exposure measurements will be captured using EasyLog USB temperature and humidity data loggers and wearable devices validated against the gold-standard Quest Temp°34 3M WBGT Monitor to calculate the WBGT heat index. The EasyLog USB data loggers will be deployed in a fixed location in the participant’s home for 24 hours to capture continuous area-level data. These devices are not worn; instead, they remain stationed in a participant’s key microenvironment throughout the monitoring period and are retrieved after for data download. Wearable devices in this study include the Fitbit Sense 2 smartwatch and iButton sensors. The Fitbit tracks maternal heart rate while the iButtons, one mounted below and another above the strap, measure the skin temperature and personal microclimate or ambient temperature, respectively. The iButtons are validated tools for capturing ambient temperature exposure in field settings and have been widely used in heat stress research ^10^. They measure temperature at 5-second intervals for 24 hours to assess individual thermal exposure and provide a low-burden, non-invasive method for continuous monitoring. Combined, these wearables provide a detailed profile of the interaction between environmental conditions and maternal physiology during daily routines. Area heat exposure will also be recorded with the Quest Temp°34 monitor. The WBGT Heat Index was selected over other indices due to its practicality and wide usage in occupational and environmental health studies. WBGT is an internationally recognised index for assessing occupational heat stress and aligns with ISO standards, particularly in settings where radiant heat and humidity are significant. The exposure data obtained will be used for the calculation of the WBGT heat index using the standard formula (ACGIH 2021). The Quest Temp°34 3M WBGT Monitor will serve as the field reference instrument. All devices will undergo pre-deployment bench checks across expected temperature and humidity ranges. The EasyLog data logger and iButton devices will be co-located with QuestTemp WBGT for ≥ 24 hours to characterise bias and precision. A rotating panel of devices will be deployed across sites quarterly to detect drift or site-specific bias. Correction equations (linear or GAM) will be derived from co-location data. Devices will be flagged for recalibration if bias means exceed 0.5°C or limits of agreement exceed ± 1.5°C; data failing QC will be excluded or imputed using measurement-error-aware methods.

Physiological heat strain will be captured via urine specific gravity measures, temperature, heart rate, and blood pressure measurements. Urinary specific gravity will be measured during a home visit in each trimester using the ATC (REC-200 ATC) clinical handheld refractometer and Dirui H10 urine dipstick. Blood pressure will be measured with the participant seated comfortably, from the brachial artery measured at the level of the heart, using an Omron HEM 7120 sphygmomanometer once per home visit and pre-shift and post-shift in working pregnant women. Core body temperature via tympanic membrane will be measured using a Rossmax RA600 infrared thermometer once per visit. Heart rate will be measured using Fitbit Sense 2 watches.

Area level heat metrics, from WBGT and Easy Log USBs, and personal exposure data from iButtons are synchronised with physiological parameters such as heart rate, core body temperature, and Urine Specific Gravity. This enables a composite understanding of external heat stress and internal physiological heat strain. All devices are time-synchronised upon starting and periodic calibration and QC procedures described above will be implemented to maintain data quality, and finally data are analysed jointly to determine how environmental conditions translate into physiological responses during pregnancy.

Quantitative data on women's perceptions of heat stress impacts, hydration, and productivity will be gathered using a modified High Occupational Temperature Health and Productivity Suppression (HOTHAPS) questionnaire. The original HOTHAPS tool was developed for occupational settings to assess heat stress impacts on health and work capacity. This version includes pregnancy-specific items and contextual modifications relevant to Indian conditions, such as household labour, rest patterns, clothing, and heat-adaptive behaviours ^6^.

To capture air pollution also exposure, indoor PM 2.5 measurements will be taken along with the heat stress parameters using an indoor monitor (Aurassure Care Real Time Personal Air Particulate Matter Monitor), calibrated to the local pollution monitoring station corresponding to the three clinical sites for a continuous period of 1 week to account for daily variations.

Quality Control (QC) monitoring for environmental exposure assessment will be done contemporaneously throughout the study using a decentralised data transfer pipeline to facilitate the cleanup and verification of the exposure assessment data collected from all three sites. This digital platform will help to limit travel needed for the exposure assessment team (based in Chennai) and will thus help to reduce our study-associated carbon footprint.

### Maternal haemodynamic assessment

It is hypothesised that the effects of heat exposure on the maternal circulation may depend on the gestational age at exposure. Specifically: (i) early pregnancy maternal hemodynamic disruptions will directly impact early placental development and increase the risk of preeclampsia and other hypertensive disorders of pregnancy, whilst (ii) mid-to-late pregnancy hemodynamic changes may influence cardiovascular load, leading to maternal cardiovascular maladaptation, which could lead to changes in the uteroplacental circulation.

Maternal hemodynamic status, including pulse, blood pressure, Cardiac output (CO), and total peripheral vascular resistance (TPVR), will be measured each trimester using the USCOM 1A® pulsed wave monitor. The USCOM 1 A is a non-invasive, portable machine that utilises continuous wave Doppler technology and it has been validated for use in pregnancy ^11^. 

All examinations will be undertaken under standardised conditions using the same equipment in each site. Women will be asked to sit quietly for 5 minutes before the commencement of the investigations. A brachial blood pressure reading will be recorded using the appropriate cuff size from the right arm of the participant. Participant height (cm) will be measured during enrolment, and weight (kg) will be recorded at the time of each assessment. Two USCOM readings will be taken at an interval of 1 minute apart, with the average of the two readings used for analysis. Participants with abnormal hemodynamic results will be referred to a hospital physician for appropriate care, facilitated by the study staff.

### Fetal and placental ultrasound and CTG assessments

It is also hypothesised that ambient heat exposure could alter Uterine Artery (UtA) flow, which in the first trimester could lead to poor trophoblast invasion, and increased risk of preeclampsia and other placental disorders ^12^, whilst later in pregnancy could alter umbilical artery Doppler readings. This reduction in perfusion could lead to lower fetal oxygen levels, causing blood redistribution to vital organs, which is detectable as changes in the middle cerebral artery (MCA) Doppler and the cerebroplacental ratio (CPR). The latter is a key predictor of perinatal morbidity and mortality in fetuses at risk for growth restriction ^13, 14^. Chronic exposure to heat with fetal blood flow redistribution could lead to reduced fetal growth ^15, 16^.

The detailed imaging protocol is reported elsewhere ^7^, but in brief the study will use trasnadominal two-dimensional ultrasound to obtain the key ultrasound parameters of the following:

At 11–14 weeks of gestation

- Crown-rump length (CRL) for pregnancy dating ^17^- conducted between 9 to <14 weeks and is used for all subsequent analyses, including preterm birth classification.

At each subsequent ultrasound assessment

- Biparietal diameter (BPD), head circumference (HC), abdominal circumference (AC) and femur length (FL) for biometry
- Estimated fetal weight (EFW)
- Amniotic fluid index easurement (AFI)
- Maternal cervical length
- Blood flow assessment using color flow Doppler of the Uterine artery at 18–22 and 30–34 weeks of gestation
- Umbilical artery at 30–34 weeks of gestation
- MCA and calculation of CPR at 30–34 weeks of gestation

In addition to the routine, detailed fetal anatomical survey performed at the second-trimester scan according to standard protocol, we will confirm that a basic structural assessment is conducted to identify any major congenital anomalies. At the same examination, placental characteristics—including location, morphology and biometric measurements—will be recorded to ensure we capture any exposure‐related vascular or placental changes.

Gestational age-based reference ranges for the pulsatility index in the umbilical artery, fetal middle cerebral artery, and the cerebroplacental ratio will be used to analyse and interpret the doppler-based placental blood flow indices. The doppler assessments will be conducted once in the third trimester (30–34 weeks of gestation) for study purposes. Repeat measurements for clinical care purposes will be as per the institutional standard of care as decided upon by the treating fetal medicine physician ^18^.

Fetal well-being is further assessed in the third trimester using non-invasive cardiotocography (CTG), which enables continuous monitoring of fetal heart rate (FHR) and uterine contractions, providing dynamic insights into fetal autonomic regulation and potential effects of heat-related stress. Abnormal CTG patterns—such as reduced variability, late decelerations, or tachycardia—may indicate fetal compromise and are evaluated alongside concurrent heat exposure data to explore potential associations.

Each participant undergoes CTG recording for at least 20 minutes during their third-trimester antenatal review to ensure sufficient data collection. CTGs are initially assessed in real-time during the participant’s visit using standard clinical protocols at each site. The CTG devices employed in the prospective study automatically analyse recordings using built-in computerised thresholds based on NICE and FIGO guidelines for fetal heart rate monitoring. If an abnormal result is detected, the clinician is alerted, prompting further assessment and monitoring of the mother and fetus by the hospital’s obstetrics team.

All CTG records are also transferred to a central database for further analysis using the Dawes-Redman algorithm, a validated and gold-standard method that calculates and extracts key FHR features and assesses them against the Dawes-Redman ten-point safety criteria. This automated approach minimises inter-observer variability, eliminating the need for additional inter-rater reliability checks or adjudication procedures.

All ultrasound procedures will follow a standardized imaging protocol and strict image quality control (QC) procedures drawing from previous similar protocols of INTERGROWTH & GARBH-INi using the same machine General Electric Healthcare (Illinois, USA) Voluson E8 machine ^7^. QC for CRL, biometry and Doppler will be conducted on 10% of images, randomly sampled, to identify any sonographers not adhering to protocol, with targeted retraining provided as needed. This QC process will be managed by an experienced team that will independently review images based on predefined criteria, ensuring high inter-rater reliability.

QC for CRL, biometry and Doppler, based on established criteria ^19– 22^, will be conducted on 10% of images, randomly sampled, to identify any sonographers not adhering to protocol, with targeted retraining provided as needed. This QC process will be managed by an experienced team that will independently review images based on predefined criteria, ensuring high inter-rater reliability.

### Placental circulating biomarkers

It is hypothesised that exposure to elevated environmental temperatures may impair placental function, leading to measurable alterations in circulating angiogenic and inflammatory biomarkers. A critical component of this dysfunction may involve changes in the molecular cargo of placental small extracellular vesicles (sEVs), which play a central role in mediating cell-to-cell communication during pregnancy ^23– 25^. Disruption of this signalling network may adversely influence maternal immune modulation, vascular function, and placental development, ultimately contributing to complications such as preterm birth, fetal growth restriction, and stillbirth. Building upon findings from the retrospective GARBH-Ini cohort (WP1a; Figure 1), this prospective study aims to validate key placental biomarkers and elucidate mechanistic pathways implicated in heat-induced pregnancy complications. Environmental temperature exposure will be quantified using high-resolution spatiotemporal models and stratified across participants from rural and peri-urban settings in India.

Maternal venous blood samples collected at predefined gestational windows will be utilized to assess the cumulative and time-specific effects of chronic heat exposure on placental physiology. Distributed lag non-linear models (DLNM) and generalized additive models (GAM) will be used to identify critical windows of gestational vulnerability to heat exposure. Based on model-derived exposure classifications, maternal samples will be stratified into high and low exposure groups to quantify surrogate markers of placental stress. Key surrogate markers of placental stress will be quantified using immunoassay-based platforms. These include angiogenic markers (sFlt-1 ^26^, PlGF, VEGF ^27^, PAPP-A ^28^), hormonal markers (progesterone), and inflammatory mediators (C-reactive protein [CRP], alpha-1-acid glycoprotein [AGP] ^29, 30^). This multi-marker approach will allow for a comprehensive assessment of placental health in the context of heat exposure. In addition, proteomic candidates previously identified in the retrospective GARBH-Ini dataset (WP1a; Figure 1) will be evaluated in this prospective cohort to confirm differential expression in response to varying levels of heat exposure and various pregnancy outcomes. Additionally, functional validation of selected protein candidates will be conducted using *in vitro* placental cell models to assess their role in stress-induced placental dysfunction.

The outcomes of this investigation are expected to deepen the mechanistic understanding of environmental heat stress on pregnancy and support the development of predictive biomarkers for early diagnosis and intervention in heat-related adverse pregnancy outcomes.

### Lactation assessment

Extreme heat exposure around birth is postulated to delay lactation onset after childbirth, reduce milk volume, and alter lactation hormones such as prolactin ^31, 32^. This sub-objective will be investigated in a targeted subset of 100 women in the lactation study (50 from the winter cohort and 50 from the summer cohort) all recruited from the GCH site. Four characteristics of lactation will be measured: (i) Timing of lactation onset assessed daily using a validated breast fullness scale during postpartum days 1–4, with a score of 3 or more indicating lactation onset ^32^; (ii) Milk maturation will be monitored daily/alternate days basis from days 1–16 by measurement of milk Na ^+^ and K ^+^ using portable ion electrodes (Horiba Na ^+^ and K+ laqua twin probes, Kyoto, Japan); (iii) The composition of colostrum, transitional milk, and mature milk will be characterised using a mid-infrared spectroscopy-based human milk analyzer, with foremilk and hindmilk nutrients being specifically assessed, and samples stored at -80°C for further analysis; (iv) Serum lactation hormones, particularly prolactin, will be measured before and after breastfeeding on postpartum days 15–16 ^33^. During the lactation assessment, at least one measurement of heat stress and PM 2.5 in the home will be captured.

### Data management

**Figure 2.**
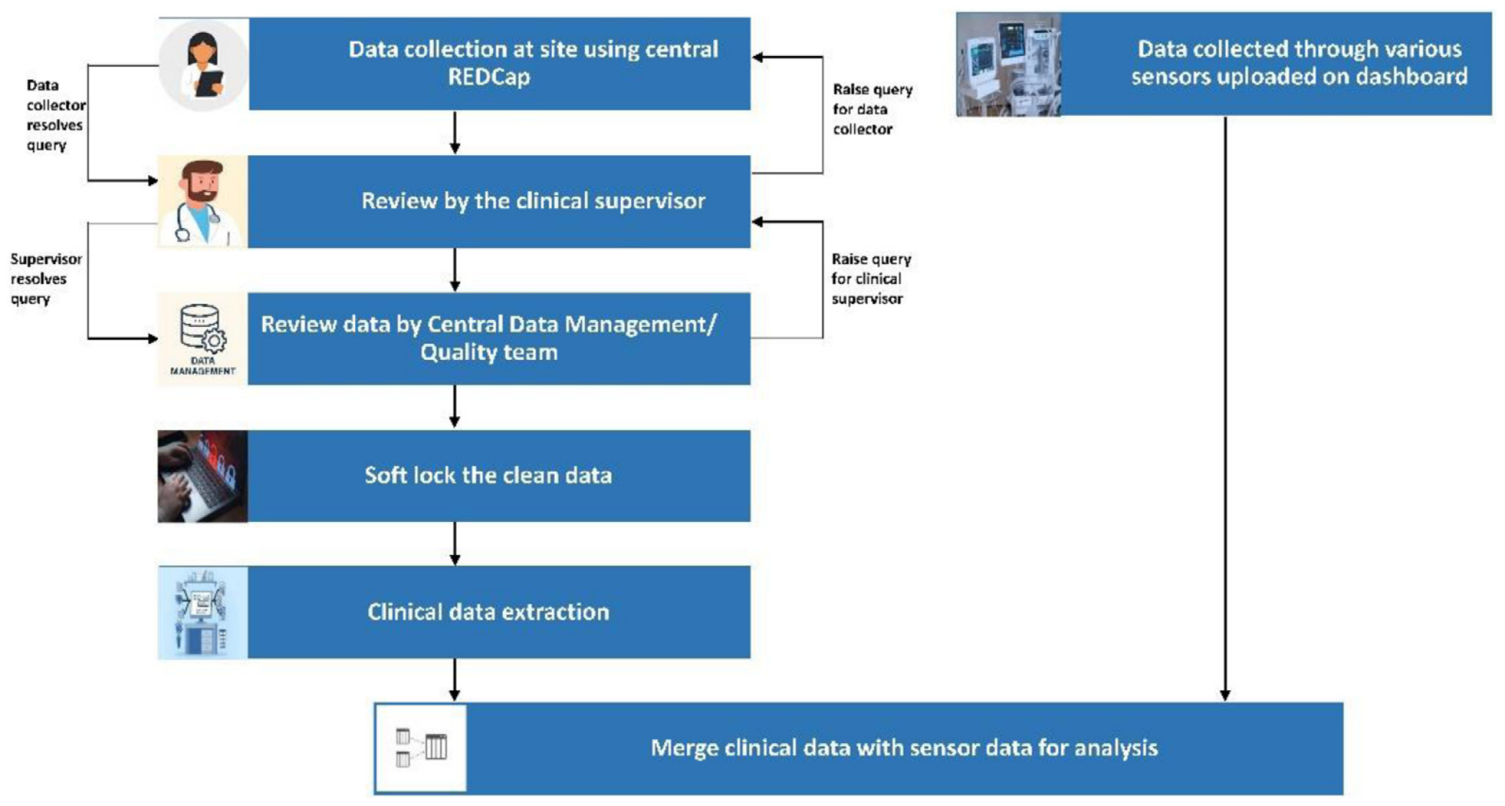
Flow of data from clinical sites to centralized Data Management Centre at THSTI.

Data will be collected using electronic case record forms (e-CRFs) on the RedCap platform using either tablet computers or smartphones, leveraging the existing GARBH-Ini data management system. The database has a two-factor authentication and does not allow the storage of data on the local device. The schematic of the data management plan is outlined below, and a detailed data management plan has been formulated ( Figure 2).

### Quality management protocol

A well-structured and detailed quality management plan has been developed, which includes of periodic monitoring activities at regular 15-day intervals both at the clinical sites as well as the home/workplace area. This is done to ensure that the quality and integrity of the data collected are in accordance with the study protocol, Standard Operating Procedures, Good Clinical Practice (GCP) guidelines, and applicable regulatory requirements. It is also intended to ensure consistency in the monitoring across all three sites. Additionally, 20% of the participants would also be contacted in a random order to inquire about the conduct of the study staff during the home visit process.

All study team members will be made familiar with and adhere to the National Ethical Guidelines for Biomedical and Health Research involving Human Participants, as well as the relevant protocol, applicable Standard Operating Procedures (SOPS), and Good Clinical Practice guidelines. The monitoring plan describes the tools to ensure the quality of the study, including that the rights, safety and well-being of the participants enrolled in the study are protected.

### Sample size

This exploratory study will be powered primarily on multiple physiological and biochemical outcomes, rather than on clinical endpoints, many of which currently lack sufficient preliminary data to enable formal power calculations at the 5% significance level.

Primary outcomes:

Physiological and biochemical measures include maternal core body temperature, urine specific gravity, maternal cardiac output (USCOM), heart rate variability, and angiogenic ratio (sFlt-1/PlGF). Clinical outcomes include preterm birth (<37 weeks of gestation) and birth weight (grams).

Secondary outcomes:

Secondary endpoints comprise gestational age (weeks), small for gestational age (SGA), stillbirth, and additional placental and fetal biomarkers.

A total of 700 pregnant women will be enrolled to account for an anticipated attrition rate of approximately 15%, yielding an expected final sample of about 600 mother–baby dyads with complete follow-up. The majority of outcomes are continuous variables, which—following transformation to approximate normality, if required—will provide 80% statistical power at a two-sided significance level of α = 0.05 to detect a minimum difference of 0.23 standard deviations between high versus low heat exposure groups. This detectable effect size lies well within accepted thresholds for clinical relevance.

### Statistical approach

The study team will implement a pre-specified Statistical Analysis Plan (SAP), finalized and published prior to database lock, to quantify associations between environmental exposures—heat, humidity, and ambient PM _2.5_—and maternal, fetal, placental, and early lactational physiological outcomes. Analyses will follow a hierarchical framework (primary, secondary, exploratory) with clearly defined endpoints to manage multiplicity. Data will undergo standardized procedures for cleaning, validation, and coding. Patterns of missingness will be described, and multiple imputation will be applied where appropriate. All model assumptions (normality, collinearity, linearity) will be evaluated systematically.

Primary Analytical Framework – Mixed-Effects Models:

Given the longitudinal and multi-site design of the HiP-India study, mixed-effects models (LME, GLMM, GAMM) will constitute the core analytic approach.

Random effects: Site-level random intercepts will account for geographic and climatic clustering across Gurugram, Puducherry, and Chhattisgarh, while subject-level random intercepts and slopes will capture individual differences in heat-response trajectories.

Serial correlation: Autoregressive structures (e.g., AR(1)) will model correlations between repeated trimester-specific measurements.

Time-varying exposures: Trimester-specific heat exposure estimates will be modeled as time-varying covariates to assess both immediate and cumulative effects.

Model flexibility: Linear mixed models will be used for continuous outcomes (e.g., physiological measures, biomarkers), generalized linear mixed models for binary outcomes (e.g., preterm birth, SGA), and generalized additive mixed models where non-linear exposure–response patterns are expected.

Complementary Distributed Lag Non-Linear Models (DLNM):

DLNM will be employed to examine delayed and non-linear exposure–response relationships across gestation.

Lag structure: Lags will extend from enrolment through outcome ascertainment, capturing both short-term (≤28 days) and longer-term (up to ~20 weeks) effects.

Basis functions: Natural cubic splines with empirically derived knots will model non-linear exposure and lag responses.

Temporal alignment: Exposure windows will be aligned primarily by calendar date to preserve seasonal context, and secondarily by gestational age to identify sensitive developmental periods. Hybrid alignment approaches will also be considered.

**Figure 3.**
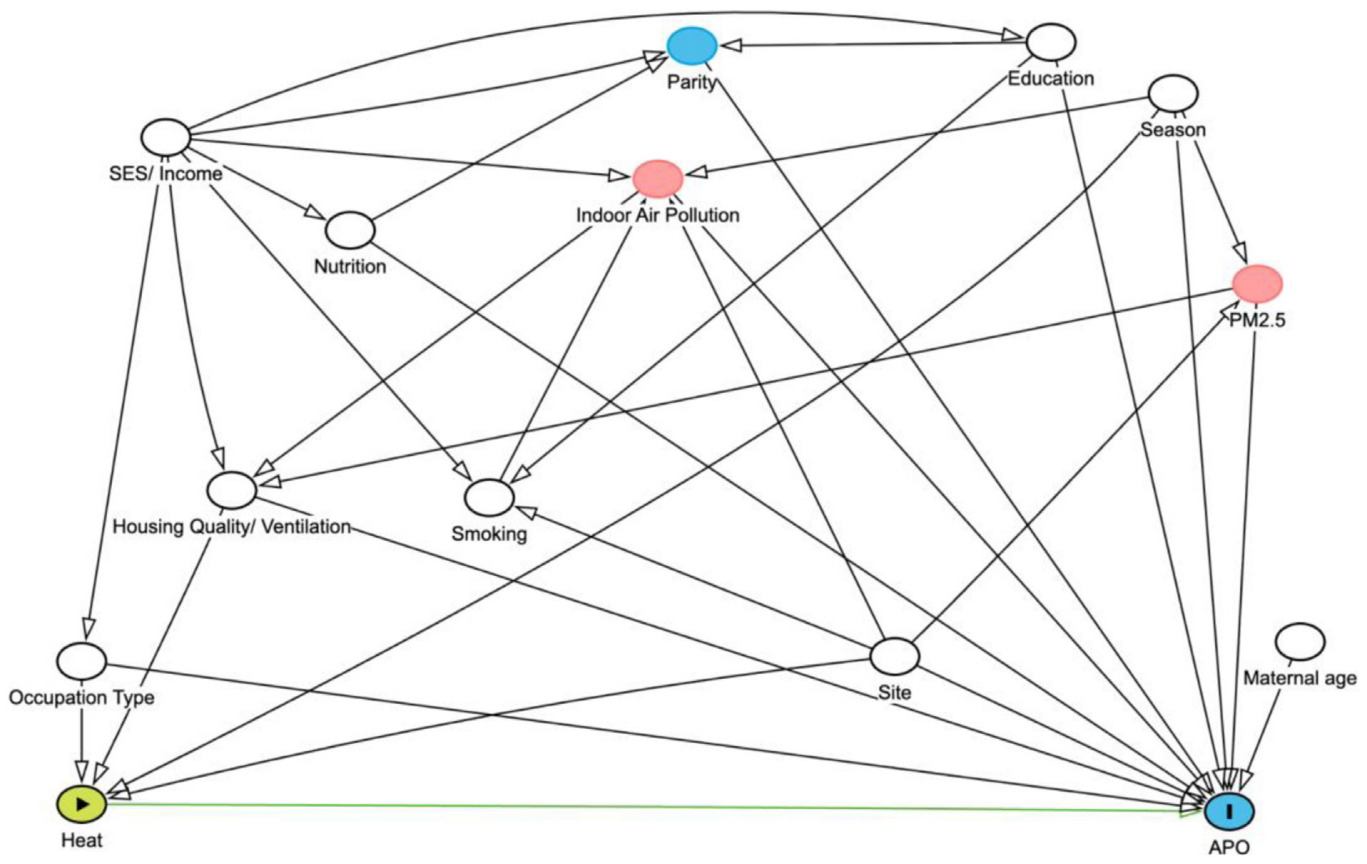
Tentative DAG to handle confounders in the analysis.

Direct Acylic Graph (DAG)-Guided Confounder Control and Effect Modification Strategy:

A tentative DAG has been developed to inform model specification and covariate adjustment ( Figure 3). The minimal sufficient adjustment set includes:

Site (geographic clustering), maternal age, education level, occupation type, family income/socioeconomic status, nutritional status, housing quality (ventilation, overcrowding), season/recruitment timing, parity, and indoor air pollution exposure (biomass fuel use, passive smoke).

PM _2.5_ Treatment Strategy:

Primary analysis: PM _2.5_ will be modeled as an effect modifier (Heat × PM _2.5_ interaction).

Secondary analysis: If significant interaction is not detected, PM _2.5_ will be adjusted as a confounder in sensitivity models.

Justification: Both exposures may exert synergistic biological influences on maternal thermoregulation and hemodynamics.

Prespecified Interactions and Stratifications:

Interactions to be formally tested include:

Heat × PM _2.5_ (synergistic effects on maternal hemodynamics), Heat × Site (climate adaptation differences), Heat × Season (acclimatization effects), Heat × Occupation (differential vulnerability by work environment), and Heat × Housing Quality (thermal protection efficacy).

Stratified analyses will be conducted by site (Gurugram, Puducherry, Chhattisgarh), recruitment season (warm vs. cool), occupation (high-risk vs. lower-risk), housing quality (traditional vs. modern materials), and gestational timing (early vs. mid vs. late pregnancy enrolment).

Mediation and Sensitivity Analysis Framework:

The study design facilitates mediation analysis through temporally aligned data collection, with personalized exposure monitoring, blood pressure measurement, biomarker sampling, and clinical assessment conducted synchronously at three points across pregnancy. This alignment strengthens the exposure → mediator → outcome temporal sequence and reduces bias from measurement misalignment.

To address unmeasured confounding, the analysis will consider negative control approaches using biomarkers or outcomes theoretically unrelated to the heat–hemodynamic pathway, supplemented by mediational E-values to quantify the magnitude of unmeasured confounding required to alter results. The multi-site structure enables leveraging geographic and seasonal variation as natural experiments to test mediation assumptions.

To correct for measurement error, data from multiple exposure and mediator sources (personal vs. area exposures, various physiological and biomarker indices) will support formal error correction procedures where feasible. Advanced methods will include longitudinal mediation models to address time-varying confounding and Monte Carlo sensitivity analyses to evaluate robustness under assumption violations. Overall, the analysis framework will balance methodological rigor with practical constraints, transparently reporting assumptions and limitations while ensuring clinically interpretable insights into heat-mediated pathways influencing pregnancy outcomes.

Integration and Synthesis:

Findings from mixed-effects, DLNM, and mediation analyses will together characterize trimester-specific, cumulative, and mechanistic pathways linking heat exposure to maternal and fetal outcomes. Model selection and validation will apply AIC/BIC criteria and cross-validation for optimal parsimony and performance. Confounder selection will remain DAG-informed, with false discovery rate (FDR) adjustments in high-dimensional biomarker analyses to control false positives.

Together, these analytic approaches will enable a nuanced understanding of how maternal heat exposure shapes physiological adaptation and pregnancy outcomes, while generating evidence to inform targeted interventions and policy frameworks aimed at protecting maternal and neonatal health in a warming climate.

## Ethics approval and study registration

The study has received ethics approval from the following independent institutional ethics committees:

1. Translational Health Science and Technology, Institute, Faridabad (THS 1.8.1/(171)
2. Gurugram Civil Hospital (GCH/EC/2021/1826/8.12.2023/28.1)
3. Chhattisgarh Institute of Medical Sciences, Bilaspur (327/C.I.M.S/I.E.C/2024)
4. Pondicherry Institute of Medical Sciences, Puducherry (RC/2024/08)
5. The George Institute for Global Health, India (Project Number 31/2023)
6. Sri Ramachandra Institute of Higher Education and Research (IEC/24/MAR/185/07)

Any important protocol modifications (e.g., changes in eligibility criteria, outcomes, or data collection methods) will be communicated to relevant parties, including the institutional ethics committee, trial registry (if registered), and study collaborators. Updated versions of the protocol will be archived and shared through the selected data repository and included in any subsequent publications.

Based on our experience with the ongoing large hospital-based pregnancy cohort at Gurugram Civil Hospital, India (GARBH-Ini) ^7^, we anticipate an overall loss to follow-up of 10–15% (study completion rate 85–90%).

Mitigation measures include:

• verifying home addresses at enrolment;

• maintaining regular contact via phone/SMS/WhatsApp;

• leveraging a field worker network for tracing and counselling;

• providing transport support or arranging home visits when hospital follow-up is not feasible.

Additional retention strategies include reimbursements for time/travel, provision of pregnancy health education materials, and facilitation of clinical care at study sites. To ensure adequate power, we will over-enrol by 15–20% to compensate for anticipated losses.

### Real-time procedures for identifying and managing hazardous heat exposure during monitoring

During the study follow-up, hospitals in our setting have established protocols for managing heat stress and heat stroke. In our study, all participants presenting with signs of heat stress (e.g., dizziness, cramps, nausea, excessive sweating, confusion, collapse) or heat stroke (e.g., high body temperature, altered mental state, confusion, hot dry skin, rapid heartbeat, nausea, headache, seizures, collapse) will be immediately referred to the hospital physician for evaluation and management as per the hospital’s standard of care.

Field staff will be trained to recognize early warning signs of heat illness, provide initial support (rest in shaded/cool areas, hydration with oral fluids), and facilitate prompt referral. Participants will also receive information on recognizing heat stress symptoms and a 24-hour contact number for emergencies. All heat-related events will be documented as part of ongoing safety monitoring.

## Discussion

The description of a novel cohort of pregnant women living in three separate locations across India, representing different climate zones is being presented in this manuscript. This cohort is one of eleven projects funded under the same call, "Biological and Physiological Effects of Extreme Heat on Pregnancy and Childhood," which is supported by Wellcome. It is anticipated that these cohorts together will advance our understanding of this critical issue as we adapt to a warming world.

Pregnant women residing in low- and middle-income regions are most susceptible, particularly in tropical environments ^34^. This study will address the lack of evidence and awareness on the impact of ambient heat on the physiological processes of mothers, fetuses, placentas, and during lactation. The team expects that the findings will have wide applicability across India and other similar countries, because of the multi-site design, which includes several temperature zones across India, such as humid subtropical and tropical Chhattisgarh, tropical Puducherry, and semi-arid Gurugram, and attention to quality control and standardisation across all study elements. This will allow us to conduct reliable and reproducible analyses of heat exposure under different environmental conditions.

Participation in this study was intentionally restricted to “high heat-risk” women without fixed air-conditioning to fulfill the primary objective of elucidating biological mechanisms linking heat exposure with adverse maternal outcomes. Including women with continuous cooling access would have reduced exposure contrast and potentially confounded estimates of heat’s direct physiological effects, as air conditioning serves as a known protective measure. By focusing on women lacking continuous cooling, the study targets the most vulnerable group—those at greatest biological and public health risk—thereby enhancing causal inference and ensuring that findings remain directly applicable to populations most in need of evidence-based heat adaptation strategies.

This study's strongest aspect is the multidisciplinary team, which brings together experts in clinical obstetrics, environmental exposures and occupational hygiene, maternal haemodynamics, fetal ultrasound, placental biology, lactation physiology and epidemiology. The work enhances the field by examining potential biomarkers and physiological indicators of heat-related stress. It also offers valuable insights into the molecular pathways that underlie the observed connections between heat exposure and adverse pregnancy outcomes. Another objective of the study is to integrate advanced technologies such as the USCOM 1A® device and portable ion conductivity devices in three climatologically distinct zones. In addition, while the study primarily focuses on heat exposure, we are also considering the critical interaction with air pollution, a major public health challenge in India.

The findings will provide a foundation for future investigations into targeted medicines, as well as guide the development of public health interventions aimed at adaptations for heat exposure during pregnancy. This study highlights the interconnectedness of maternal health, environmental health, and climate change, and underscores the pressing need for adaptive policies to protect vulnerable populations from the impacts of increasing global temperatures.

## Patient consent

Written informed consent will be obtained from all study participants before enrollment. Informed consent will be obtained in person by trained research staff at site. Staff will explain the study objectives, procedures, and risks in the local language, and participants will be given time to ask questions before signing the consent form. All personal information will be collected using encrypted tablets and stored on secure, password-protected servers. Identifiable data will be accessible only to authorized study personnel. De-identified datasets will be used for analysis and sharing, ensuring confidentiality is maintained throughout and after the study.

## Study registration- clinical trial registry

The name of the trial is Effects of extreme heat on maternal, placental and fetal physiology, lactation and newborn health in India. The study has been registered with the Clinical Trials Registry of India (CTRI)- (CTRI/2024/12/078527). Registered on 24/12/2024 and it will open for 3 years.

## Protocol version

Protocol version 10, dated e.g., 26 August 2025

## Structured summary

- Public title: Heat in Pregnancy – India
- Scientific title: Effects of extreme heat on maternal, placental and fetal physiology, lactation and newborn health in India
- Study design: Prospective cohort study
- Study population: Pregnant women enrolled in the first trimester in three sites: Gurugram, Chhattisgarh, Pondicherry
- Setting: Mixed hospital and community-based recruitment sites in Gurugram, Chhattisgarh, Pondicherry during winter and summer months
- Target sample size: 600 participants (300 in the summer group and 300 in the winter group)
- Intervention/exposure: Natural seasonal heat exposure (participants recruited in summer vs. winter months)
- Comparator/control: Women recruited in winter months serve as the comparator group
- Primary outcomes: Incidence of preterm birth (<37 weeks); birth weight
- Secondary outcomes: Gestational age, stillbirth, and other maternal/newborn health outcomes
- Key inclusion criteria: Pregnant women in 11–14 weeks, aged 18–45 years, residing in the area for at least 6 months
- Key exclusion criteria: Women with pre-existing chronic illnesses or those unlikely to remain in the area during follow-up
- Study period: Expected recruitment from May 2023 to March 2026

Ethics approval: Approved by

1. Translational Health Science and Technology, Institute, Faridabad (THS 1.8.1/ (171)
2. Gurugram Civil Hospital (GCH/EC/2021/1826/8.12.2023/28.1)
3. Chhattisgarh Institute of Medical Sciences, Bilaspur (327/C.I.M.S/I.E.C/2024)
4. Pondicherry Institute of Medical Sciences, Puducherry (RC/2024/08)
5. The George Institute for Global Health, India (Project Number 31/2023)
6. Sri Ramachandra Institute of Higher Education and Research (IEC/24/MAR/185/07)
• Trial registration: Clinical Trials Registry of India (CTRI)-(CTRI/2024/12/078527), registered on 24/12/2024 open for 3 years.
• Data sharing: De-identified participant data and metadata will be shared through Zenodo, a public repository: **10.5281/zenodo.16421773**
• Funding source: Supported by the Wellcome Trust

### Glossary terms

Heat (or thermal) stress: Ineffective dissipation of metabolic heat in hot environments and/or during physical exertion or exercise ^35^.

Ambient heat exposure: The exposure of an individual or population to elevated outdoor environmental temperatures, typically measured through air temperature, radiant temperature, humidity, and wind speed ^9^.

Threshold Limit Value: Threshold Limit Value (TLV) is defined by the ACGIH as the level of heat stress exposure, measured by Wet Bulb Globe Temperature, to which nearly all acclimatised, adequately hydrated, healthy workers may be exposed without adverse health effects ^9^.

Physiological heat strain: The effect of environmental heat stress on the body ^36^.

## Figures and Tables

**Table:** 

Institute	Investigators
Translational Health Science and Technology Institute (THSTI)- Faridabad, Haryana, India	Shinjini Bhatnagar Nitya Wadhwa Pallavi Kshetrapal Shailaja Sopory Ramachandran Thiruvengadam Bapu Koundinya Desiraju Savita Singh Neera Parmar Dharmendra Sharma Ayushi Sreevatsan Raghavan Lovejeet Kaur Mudita Gosain Sumit Misra Prakriti Dayal Divya Arya Mukesh Juyal Taruna Rattan Rajkumar Tanwar Shilpa Chopra
The George Institute for Global Health, Imperial College, London, UK	Jane Hirst Mark Woodward Gabriela Cipriano Flores
Pondicherry Institute of Medical Sciences, Puducherry, India	Stalin Prabakaran Ramachandran Thiruvengadam Mary Daniel Padma A Sunil Kumar Nanda Joseph Manuel Manikandan M Roselin M Saranya R Velavan A Arya TS
The George Institute for Global Health, New Delhi, India & Chattisgarh Institute of Medical Sciences, Bilaspur, India	Yogesh Jain D Praveen Chris Marie Kurian Karthika Kumar Diana Thomas Sudhakar Reddy Bulla CIMS, Bilaspur: Sangeeta Raman Jogi Rachna Jain Prashant Nigam
Sri Ramachandra Institute of Higher Education and Research, Chennai, India	Vidhya Venugopal Rekha Shanmugam Tanya Isaac Nikitha D
Nuffield Dept of Women’s & Reproductive Health, University of Oxford, UK	Aris Papageorghiou Manu Vatish Fadil M. Hannan Gabriel Jones Julie Abraham Katherine Dibbon
Department of Atmospheric Science, Indian Institute of Delhi (IIT), Delhi, India	Sagnik Dey Kavya Bhatt Arunangsu Das Vineetha Vincent Sofiya Rao
CareMother, Mumbai, India	Shantanu Pathak
St Georges University London, UK	Basky Thilaganathan
The Ultrasound Lab, Delhi, India	Ashok Khurana

## Data Availability

No data associated with this article. Our data is available in Zenodo: **10.5281/zenodo.16421773**
