# Supplementary material for: Cohort Profile for the Heat in Pregnancy- India (HiP-India) Study
Source: Wellcome Open Res. 2025 Nov 17;10:472. Originally published 2025 Aug 29. [Version 2] doi: 10.12688/wellcomeopenres.24393.2 (PMC12854206; doi:10.12688/wellcomeopenres.24393.2)
Supplement: Peer review history

# Reviewer report: Reviewer response for version 2

**DOI:** 10.21956/wellcomeopenres.27866.r141796

**Published:** 2026-01-10

**Authors:** Darshnika Lakhoo

**Affiliations:** 1 University of the Witwatersrand, Johannesburg, South Africa

Thank you for the opportunity to review this manuscript. This is a relevant, well-designed and rigorously described multicentre protocol addressing the physiological effects of extreme heat exposure on pregnancy in LMIC settings. The proposed study integrates environmental and individualised monitoring, biological measures, fetal imaging, biomarker testing, and a novel lactation sub-study across three climatic regions. The multidisciplinary team, attention to QC, and intention to link mechanistic and clinical outcomes significantly strengthens the work. The manuscript is clear, logically structured, and improved through prior revisions.

 Given the recruitment timeline (to March 2026), some suggestions may no longer be feasible to implement. However, clarification of the points below remains important for transparency, and for informing future studies.

- There is evidence to suggest that adolescents (<18 years) are at increased risk of heat related adverse outcomes. Could the authors justify why this high-risk group is excluded?
- Are home births anticipated at any of the three sites? If so, how will this be handled? Will these participants remain in the cohort and how will outcome data be obtained?  
- In keeping with GCP, informed consent should be ongoing, rather than a once-off event as prescribed in your study schedule. Could the authors confirm if participants will reaffirm consent at each visit, even if a brief recorded confirmation?  
- Could the authors justify the absence of intrapartum or postpartum measurements such as hydration markers or maternal physiology? Given that heat stress is likely highest around delivery, and dehydration may affect lactation onset, this appears to be a missed opportunity.
- The ethics of sampling 5 ml of colostrum on postpartum day 1–2 are unclear, particularly for primiparous women who may produce very small volumes. Could the authors clarify whether smaller volumes were considered, or whether sampling might exclude those reporting low supply?
- As Reviewer 1 highlighted, a single 24-hour exposure measurement per trimester risks being unrepresentative. It remains unclear why this approach was selected and how it will be triangulated with other exposure sources. Which measurements will take precedence if devices disagree, and how will data loss be handled?
- Wearable devices may introduce safety concerns. Could the authors indicate whether there are mitigation strategies for device-related theft or stigma, and whether replacement devices will be provided if lost or stolen?
- It is unclear which pregnancy outcomes will be actively collected versus extracted from routine care records (e.g., gestational diabetes, hypertension in pregnancy, anaemia, HIV). Is there standardisation across sites for diagnosis and documentation of these outcomes?
- The manuscript references sensitivity analyses based on “gestational timing of exposure,” yet all participants are enrolled at 11–14 weeks. Could the authors clarify what variation is expected? Similarly, what is meant by “gestational age” as an outcome?
- Describing winter-recruited participants as a comparator group may be misleading, as most pregnancies traverse both hot and cooler periods. I suggest reframing as “lower exposure periods” rather than a formal comparator group.
- Finally, and in keeping with Reviewer 1, the hierarchy of primary and secondary outcomes remains inconsistent between the main text and the structured summary. Could the authors harmonise these definitions across the document and trial registration?

Is the study design appropriate for the research question?

Yes

Is the rationale for, and objectives of, the study clearly described?

Yes

Are sufficient details of the methods provided to allow replication by others?

Partly

Are the datasets clearly presented in a useable and accessible format?

Not applicable

Reviewer Expertise:

Clinical medicine, climate change and health, maternal health and climate change, infectious diseases, public health

I confirm that I have read this submission and believe that I have an appropriate level of expertise to confirm that it is of an acceptable scientific standard.

# Reviewer report: Reviewer response for version 2

**DOI:** 10.21956/wellcomeopenres.27866.r141801

**Published:** 2026-01-09

**Authors:** Srishti Sadhir

**Affiliations:** 1 Institute for Biospheric Studies & Department of Anthropology, Yale University (Ringgold ID: 5755), New Haven, Connecticut, USA

This article presents the protocol for a prospective cohort study on maternal and fetal physiological responses to extreme climate conditions, particularly high heat exposure, across India. The planned multicenter study spans three locations that differ by climate zone. The distinct climate dynamics of each location are well-defined and differentiated from one another, which is a limitation of existing studies that often lump several distinct climate zones under one "high-heat" umbrella. Several techniques will be employed to collect environmental variables, from macro- to micro- to physiological scale (i.e., heart rate and body temperature). Physiological and psychosocial data will be collected related to heat strain and perception of heat stress. Recruitment criteria and data collection schedules are also clearly defined. Statistical approaches are appropriate to address study hypothesis and objectives.

 The authors have a clear focus on biological mechanisms and specific physiological markers that might mediate the relationship between climate conditions and pregnancy/postpartum outcomes. Of particular focus is maternal haemodynamics, fetal and placental anatomy and physiology, placental biomarkers, and milk assessment in postpartum. The authors present a detailed, comprehensive set of physiological markers to be measured that will contribute profoundly to our knowledge of physiological responses during pregnancy and lactation.

 While I approve this article, I present a few points the authors may consider when implementing this study. Firstly, is there an expectation to find underlying demographic and socioeconomic differences between the three populations that might affect outcomes independent of climate variables? Even if recruitment is based on elevated risk of heat exposure via occupational or household factors, I would expect there to be more specific differences that could drive differential outcomes. It is good to see that demographic and socioeconomic variables will be included in the statistical models, but I'd like to see some discussion about possible known population-level differences in lifestyle or circumstance, as well as prevalence of adverse pregnancy outcomes, between the three populations.

 Since recruitment will occur well into the first trimester--which is a valid strategy given the scope and scale of this study--I suggest the authors consider the impact of maternal preconception condition and exposure driving pregnancy outcomes. Recruitment for this study will occur in the warm season for each location, but conception would have occurred in the previous cool season, approximately 2-3 months prior. Recent pregnancy physiology literature has highlighted the importance of maternal condition before conception shaping pregnancy trajectories, particularly in terms of macronutrients (see for example, Kuzawa, 2020; Thayer et al., 2020). I do not think the chronic vs. acute effects of extreme climate exposure are well understood in the context of pregnancy and postpartum, but these effects may also be working on different timescales. It may be appropriate to consider lagged variables or models (as already outlined in the statistical approach section) in which the macro-climate conditions are extracted for the season in which conception occurred, for example. While micro-climate conditions would not be available, existing macro-climate data might provide some insight into maternal preconception effects, as a tertiary objective.

 Overall, this study is novel in its distinct focus on biological mechanisms, and its clinical relevance for managing health concerns in a changing world. I look forward to reading indices resulting from this study in the future.

Is the study design appropriate for the research question?

Yes

Is the rationale for, and objectives of, the study clearly described?

Yes

Are sufficient details of the methods provided to allow replication by others?

Yes

Are the datasets clearly presented in a useable and accessible format?

Yes

Reviewer Expertise:

I am a physiologist and maternal health researcher studying energy balance, physical activity, body composition, and health outcomes during pregnancy. I am interested in how women manage the energy costs of pregnancy given diverse lifestyles and environments. I focus primarily on metabolic outcomes, and have published on the effects of environmental and lifestyle exposure on pregnancy physiology. I utilize stable isotope analysis, indirect calorimetry, accelerometry, and enzyme-linked immunosorbent assays as part of my methodological approach.

I confirm that I have read this submission and believe that I have an appropriate level of expertise to confirm that it is of an acceptable scientific standard.

## References

[1] : Pregnancy as an intergenerational conduit of adversity: how nutritional and psychosocial stressors reflect different historical timescales of maternal experience. *Current Opinion in Behavioral Sciences* .2020;36: 10.1016/j.cobeha.2020.07.001 42-47 10.1016/j.cobeha.2020.07.001

[2] : The Maternal Nutritional Buffering Model: an evolutionary framework for pregnancy nutritional intervention. *Evolution, Medicine, and Public Health* .2020;2020(1) : 10.1093/emph/eoz037 14-27 10.1093/emph/eoz037 PMC699044832015877

# Reviewer report: Reviewer response for version 2

**DOI:** 10.21956/wellcomeopenres.27866.r139895

**Published:** 2025-11-22

**Authors:** Huiqi Chen

**Affiliations:** 1 London School of Hygiene & Tropical Medicine, London, UK

Thanks to the authors. The revisions have substantially strengthened the protocol description and adequately addressed main concerns raised in my previous report.

 I have no further substantive comments. Only minor editorial issues remain (e.g. occasional typo errors such as “plancental” instead of “placental”). I am happy to recommend approval of this revised version.

Is the study design appropriate for the research question?

Yes

Is the rationale for, and objectives of, the study clearly described?

Yes

Are sufficient details of the methods provided to allow replication by others?

Partly

Are the datasets clearly presented in a useable and accessible format?

Not applicable

Reviewer Expertise:

Environmental epidemiology; temperature–health relationships (heatwaves, high temperature); exposure assessment & time-series/multi-location modelling; maternal–fetal and perinatal health outcomes.

I confirm that I have read this submission and believe that I have an appropriate level of expertise to confirm that it is of an acceptable scientific standard.

# Reviewer report: Reviewer response for version 1

**DOI:** 10.21956/wellcomeopenres.26897.r131207

**Published:** 2025-09-16

**Authors:** Huiqi Chen

**Affiliations:** 1 London School of Hygiene & Tropical Medicine, London, UK

This is a well-conceived, multicentre prospective cohort on heat exposure in pregnancy spanning three climatic zones in India. It integrates home/workplace environmental monitoring, wearable physiological measures, standardized fetal ultrasound and Doppler, and maternal biomarkers, with rigorous QC and data governance. The mechanistic framing, from exposure to maternal haemodynamics and placental function to fetal blood flow, growth, and perinatal outcomes, is clear and of public-health and translational relevance. However, there are important gaps in outcome hierarchy and power alignment, exposure representativeness, repeated-measures statistical framework, and issues of ethical handling.

 Major comments

 1. Primary outcomes and power alignment. The main text positions physiological/biochemical endpoints as the “primary” focus, whereas the structured summary lists preterm birth and birth weight as primary outcomes; the sample size is powered for continuous measures (~0.23 SD). Please harmonise the outcome hierarchy (primary/secondary) across the manuscript and registry, and provide power or minimum detectable effects for each primary outcome, including assumed event rates and attrition.

 2. Exposure representativeness and measurement error. A single 24-h assessment per trimester may not represent trimester-level exposure, and multiple instruments (QuestTemp WBGT, EasyLog/iButtons/Fitbit) can introduce systematic differences. Please justify the 24-h strategy and could add a small repeated-measurement sub-study (e.g., ≥48–72 h, or two 24-h sessions) to estimate within- and between-day variance for regression calibration or explicit error models. Describe field calibration against the gold-standard WBGT, cross-site drift control, and how discrepant devices will be reconciled. Clarify the rationale and limits of applying ACGIH TLVs to the general (non-occupational) population, and preferrably perform sensitivity analyses using alternative indices (e.g., UTCI, Heat Index?).

 3. Statistical framework for repeated measures. Given clustered, longitudinal data, mixed-effects models (LME/GLMM/GAMM) should be the primary framework to account for site- and subject-level random effects and serial correlation. For DLNMs, specify the lag structure, knot placement, and how gestational time is aligned (calendar time vs gestational week) to avoid temporal confounding.

 4. Confounding and effect modification. Use a DAG to define the minimal sufficient adjustment set (e.g., anaemia/nutrition, workload/physical activity, housing/ventilation, rest/hydration, socioeconomic factors). Clarify whether PM2.5 is treated as a confounder or an effect modifier and prespecify key interactions and stratifications (site, season, occupation, housing materials, fetal sex).

 5. Restricting to “high heat-risk” women and excluding those with fixed air conditioning may inflate effect estimates and limit external validity. Please acknowledge and, if possible, quantify this limitation.

 6. Outcome and QC. Preterm birth classification should state how CRL-based dating is reconciled with later GA assessments and thresholds for re-dating. Define abnormal thresholds and repeat-measurement rules for Dopplers and placental indices, and describe CTG interpretation procedures, inter-rater reliability checks, and adjudication.

 7. Mediation analysis identifiability. The proposed mediation pathway ('heat → haemodynamics/placenta → fetal growth/outcomes') requires strong assumptions. Please outline approaches to address unmeasured mediator–outcome confounding and exposure/mediator measurement error, and discuss the feasibility of negative controls or instrument-based strategies.

 8. Ethical management of high risk. Please provide expected loss-to-follow-up and adherence rates with mitigation plans. In the ethics section, detail real-time procedures for identifying and managing hazardous heat exposure during monitoring.

Is the study design appropriate for the research question?

Yes

Is the rationale for, and objectives of, the study clearly described?

Yes

Are sufficient details of the methods provided to allow replication by others?

Partly

Are the datasets clearly presented in a useable and accessible format?

Not applicable

Reviewer Expertise:

Environmental epidemiology; temperature–health relationships (heatwaves, high temperature); exposure assessment & time-series/multi-location modelling; maternal–fetal and perinatal health outcomes.

I confirm that I have read this submission and believe that I have an appropriate level of expertise to confirm that it is of an acceptable scientific standard, however I have significant reservations, as outlined above.
